# DO SERIOUS HEALTH EVENTS CHANGE HOW WE VIEW OUR OWN AGING? ON THE ROLE OF CARDIOVASCULAR EVENTS

**DOI:** 10.1093/geroni/igz038.2897

**Published:** 2019-11-08

**Authors:** Julia K Wolff, Svenja M Spuling, Ann-Kristin Beyer, Maja Wiest, Susanne Wurm

**Affiliations:** 1 Friedrich-Alexander-Universität Erlangen-Nürnberg, Nuremberg, Bayern, Germany; 2 German Centre of Gerontology, Berlin, Berlin, Germany; 3 Charité - Universitätsmedizin Berlin, Berlin, Berlin, Germany; 4 Freie Universität Berlin, Berlin, Berlin, Germany

## Abstract

Several studies have demonstrated beneficial effects of views on aging (VoA) on health, while the reverse relationship is seldom in focus. Serious health events (e.g., myocardial infarction) are life-threatening and remind individuals of the finitude of life possibly changing their VoA. The present study investigates the effect of cardiovascular events (CVE) on longitudinal changes in VoA using pooled data of three waves of the German Ageing Survey (2008, 2011, 2014, age-range: 40-95 years). To account for alternative explanations, individuals without CVE were matched to the individuals with CVE (n = 202) using a propensity-score-matching approach. Individuals who experienced a CVE showed more adverse changes in three VoA indicators (aging associated with physical losses, ongoing development, felt age) than individuals without CVE. Results show that CVE can change how we view our own aging which in turn affects future health changes. Following a CVE people may benefit from promoting positive VoA.

